# Novel Druggable Hot Spots in Avian Influenza Neuraminidase H5N1 Revealed by Computational Solvent Mapping of a Reduced and Representative Receptor Ensemble

**DOI:** 10.1111/j.1747-0285.2007.00614.x

**Published:** 2008-02

**Authors:** Melissa R Landon, Rommie E Amaro, Riccardo Baron, Chi Ho Ngan, David Ozonoff, J Andrew McCammon, Sandor Vajda

**Affiliations:** 1 Bioinformatics Graduate Program, Boston UniversityBoston, MA 02215, USA; 2 Department of Chemistry & Biochemistry and Department of Pharmacology and NSF Center for Theoretical Biological Physics (CTBP), University of California San DiegoLa Jolla, CA 92093-0365, USA; 3 Department of Biomedical Engineering, Boston UniversityBoston, MA 02215, USA; 4 School of Public Health, Boston UniversityBoston, MA 02218, USA; 5 Howard Hughes Medical Institute, University of California San DiegoLa Jolla, CA 92093-0365, USA

**Keywords:** computational solvent mapping, ensemble-based drug design, H5N1, hot spot, molecular dynamics, neuraminidase, receptor flexibility, RMSD clustering

## Abstract

The influenza virus subtype H5N1 has raised concerns of a possible human pandemic threat because of its high virulence and mutation rate. Although several approved anti-influenza drugs effectively target the neuraminidase, some strains have already acquired resistance to the currently available anti-influenza drugs. In this study, we present the synergistic application of extended explicit solvent molecular dynamics (MD) and computational solvent mapping (CS-Map) to identify putative ‘hot spots’ within flexible binding regions of N1 neuraminidase. Using representative conformations of the N1 binding region extracted from a clustering analysis of four concatenated 40-ns MD simulations, CS-Map was utilized to assess the ability of small, solvent-sized molecules to bind within close proximity to the sialic acid binding region. Mapping analyses of the dominant MD conformations reveal the presence of additional hot spot regions in the 150- and 430-loop regions. Our hot spot analysis provides further support for the feasibility of developing high-affinity inhibitors capable of binding these regions, which appear to be unique to the N1 strain.

The avian influenza virus type A, especially subtype H5N1, is becoming the world's largest pandemic threat because of its high virulence and lethality in birds, quickly expanding host reservoir, and high rate of mutation (1). The virus functions in combination with two major membrane glycoproteins: hemagglutinin (HA) and neuraminidase (NA), which together play important roles in the interactions with host cell surface receptors. While HA mediates virion (i.e. viral particle) entry into the cell, NA facilitates viral shedding by cleaving the sialic acid linkage formed between the HA and sialic receptors on the surface of the host cell (2). Several approved anti-influenza drugs effectively target NA, which cleaves these terminal sialic acid residues and facilitates the release of viral progeny from infected cells (3). However, antigenic drift has given rise to new strains that are resistant to existing NA inhibitors, and antigenic shift is resulting in new virulent subtypes of the flu virus, underscoring the need to design novel NA and HA inhibitors that can be combined for optimal prophylaxis and treatment.

Neuraminidase enzymes are phylogenetically categorized into two groups: group-1 includes N1, N4, N5, and N8 and group-2 includes N2, N3, N6, N7, and N9 (4). Although active site residues are largely conserved across both the groups, different NA subtypes exhibit varied drug susceptibility (5) and resistance profiles (6,7). Currently available NA inhibitors, including oseltamivir (marketed as Tamiflu, Roche Pharmaceuticals, Basel, Switzerland) and zanamivir (marketed as Relenza, GlaxoSmithKline, Brentford, London, UK), have been designed against crystal structures of group-2 enzymes [Ref. (3) and references therein]. Tamiflu, which has been stockpiled by many nations in efforts to avert a possible pandemic, is the only orally available drug that is effective against H5N1; therefore, additional drug discovery efforts against this enzyme are exceptionally well-motivated.

The first crystal structures of a group-1 NA in apo form and in complex with currently available drugs (8) revealed that although the binding pose of oseltamivir (Tamiflu) was similar to that reported in previous crystallographic complexes (9,10), the 150-loop adopted a distinct conformation, opening a new cavity adjacent to the active site. Under certain crystallization conditions, however, the 150-loop adopted the same closed conformation as previously seen in group-2 NA structures, suggesting that a slow conformational change may occur upon inhibitor binding (8). Importantly, previously published group-2 NA structures have only reported the 150-loop in the closed conformation (9,10). Therefore, the new structural observation of the 150-loop in the open conformation by Russell *et al.* (8) was postulated to be able to be exploited in the development of more effective drugs against N1. However, standard X-ray crystallography experiments only provide a single snapshot of the structure, making the interpretation of dynamical properties a difficult task and naturally motivating further studies that are able to investigate and characterize dynamics.

Molecular dynamics (MD) simulation is a useful theoretical tool to study the properties of biomolecular systems with atomic resolution based on classical mechanics (11–13). They complement experimental results by providing distributions and time series of any physically definable observable, within force field accuracy and finite sampling limitations. Extensive all-atom explicit solvent MD simulations of the apo and oseltamivir-bound (i.e. holo) systems revealed that the 150-loop and adjacent binding-site loops may be even more flexible than observed in the crystal structures (14). In the apo simulations, the 150-loop was observed to open to a greater extent than in the X-ray structures, and its motion was often coupled to an outward movement of the adjacent 430-loop. These coupled motions significantly expanded the active site cavity, increasing its solvent-accessible surface area as compared with both open and closed crystal structures. It was postulated that topological changes and additional expansion of the N1 inhibitor-binding pocket revealed by the MD simulations could potentially play an important role in the rational design of inhibitors for N1 (14).

In this work, we present new druggable hot spots, i.e. binding sub-regions that account principally for the ligand-binding energy, revealed in the N1 MD simulations by computational solvent mapping (CS-Map) (15,16). Originally developed for binding-site identification, the CS-Map algorithm replicates the experimental Multiple Solvent Crystal Structures (MSCS) method developed by Mattos *et al*. (17,18), where it was shown that consensus binding locations of co-crystallized solvent molecules are highly predictive of binding regions. Using only X-ray structures as input, numerous studies utilizing CS-Map have shown that the method accurately reproduces the results of MSCS experiments in addition to identifying important binding features for other well-studied proteins. The CS-MAP algorithm differs from similar methods such as GRID (19) and MCSS (20) in three major respects: (i) the initial rigid body search provides better sampling of protein surface regions with favorable electrostatics and desolvation than grid search or local minimization; (ii) the scoring potential accounts for desolvation and ligand flexibility; and (iii) the docked ligand positions are clustered and the clusters are ranked on the basis of their average binding affinity. Importantly, this allows us to capture consensus sites rather than the binding sites of individual probes. The combination of these elements yields an algorithm that shows remarkable robustness against variations in the protein structure and changes in energy parameters (16).

In a recent study performed by Landon *et al*. (21), CS-Map was applied to identify hot spots within druggable protein binding regions and showed excellent agreement with biophysical experiments, where the authors were able to successfully use CS-Map to identify hot spots for a range of important pharmaceutical targets. The study highlighted the accurate identification of a novel hot spot within the peptide-binding region of renin that was recently discovered at Novartis (Novartis, Basel, Switzerland) (22). After more than 20 years of research efforts, the discovery of this hot spot led directly to the development of the first orally bioavailable FDA-approved renin inhibitor. The same CS-Map-based hot spot study reported excellent correspondence between the hot spots predicted for the FK-506 binding protein and ketopantoate reductase to those originally identified by NMR (23) and isothermal titration calorimetry experiments (24), respectively. The authors of this study concluded that CS-Map is a valuable complement for biophysical experiments, in which the data resulting from mapping studies can be used to design and conveniently select drug discovery experiments.

Here, we apply CS-Map to identify additional hot spots within the sialic acid binding region of N1 using MD-generated ensembles as input. The application of CS-Map in conjunction with MD simulations is conceptually similar to the so-called ‘dynamic pharmacophore’ technique originally developed by Carlson *et al*. (25), which was one of the first experimentally verified methods to account for receptor flexibility in structure-based drug design through the use of MD simulations. This technique has been extended by the Carlson group to include the use of structural ensembles derived via NMR and X-ray crystallography, termed the ‘multiple protein structure models’ approach (26). Although both the dynamic pharmacophore method and the one described herein utilize small molecule probes to investigate the effects of structural flexibility on ligand binding, the CS-Map technique has the advantage of being directly comparable with MSCS experiments. As emphasized by Mattos and Ringe (15), the major problem with approaches exemplified by GRID (16) and MCSS (17), is that they result in too many energy minima on the surface of the protein, rendering it difficult to determine which of the minima are actually relevant. A careful study comparing the results of mapping calculations with X-ray structures of thermolysin determined in four different organic solvents (27) shows that both GRID and MCSS find minima close to the experimentally observed positions, but the closest minima are generally not among those with the lowest free energies, resulting in false positives (i.e. conformations with favorable energy which are not located near any experimentally observed binding site). However, it has been recently reported that re-ranking MCSS results with a potential that accounts for solvation improves agreement with experimental data (28).

The work we present here also combines a root-mean-square difference- (RMSD-) clustering algorithm to reduce the structural redundancy in the MD ensemble. Previous studies tackled this same computational issue using different algorithms (14,29,30). However, we note that the present study is based on considerably longer MD trajectories – equivalent of 160 ns – therefore providing an extensive sampling of the receptor configurational space. The combination of techniques in this work presents an efficient method to map target hot spots based on a reduced and representative ensemble of receptor structures, and it represents a novel application of existing computational methods for the investigation of protein–ligand interactions in the presence of significant conformational flexibility within a ligand-binding site.

The RMSD-clustering analysis of the explicitly solvated 40-ns tetramer MD simulations yielded a set of 10 structures representing the apo ensemble and 5 structures representing the holo ensemble. A series of mapping analyses were subsequently performed on the representative snapshots. The results suggest that low-energy hot spots within the 150- and 430-loop regions, determined by consensus binding positions of fragment-sized molecules, may provide additional opportunities for drug discovery that could potentially mitigate the effects of drug resistance. Furthermore, this study tackles two major methodological issues. First, it demonstrates the benefit role of employing MD simulations to capture receptor flexibility. Second, it provides a framework to apply CS-Map in conjunction with reduced and representative MD receptor ensembles to predict the binding of molecules within flexible regions with increased accuracy and efficiency.

## Methods

### Apo system setup

The crystal structures used in this study were 2HU0 for the oseltamivir-bound system and 2HTY for the apo system (8). Protonation states for histidine residues were defined at an apparent pH 6.5 using the PDB2PQR web server (31). All crystallographically resolved water oxygen positions were retained in the apo system, as well as Ca^2+^ ions, which are required for optimal NA function (32). To mimic future experimental inhibition assay conditions, a 20-mm NaCl salt bath was introduced. The simulated system contained 112 311 atoms. Additional details on the simulation setup can be found elsewhere (14).

### Holo (i.e. oseltamivir-bound) system setup

The 2HU0 structure had a single oseltamivir molecule bound in the active site of chain B (8). To introduce the oseltamivir within each of the other chains, chain B was aligned to chain A, C, and D by superimposition of the backbone C_α_-atoms and the resulting transformation matrix was also applied to the oseltamivir molecule. As no water molecules or ions were reported in the 2HU0 structure, we structurally aligned the 2HTY and 2HU0 systems and kept all crystallographic water molecules that did not clash with oseltamivir in the binding pocket. The Ca^2+^ ions were also retained as they are required for proper function. Amber9 was used to set up the oseltamivir-bound system using an identical protocol to the apo system (see earlier), with the exception of the additional oseltamivir parameters. Oseltamivir was parameterized as described in Ref. (14). The composite tetrameric oseltamivir-bound system, comprising one oseltamivir molecule in each of the four active sites and calcium ions in 20-mm NaCl salt bath, contained 112 457 atoms.

### Molecular dynamics simulations

The energy of apo and holo systems were minimized for 5 × 10^4^ steps using namd version 2.6b1 (http://www.ks.uiuc.edu/Research/namd/) (33). During the first phase of the minimization, only hydrogen atoms were relaxed for 5 × 10^3^ steps, holding all other atoms fixed. Hydrogen atoms, water molecules, and ions were relaxed during additional 5 × 10^3^ steps. In the last cycle, the protein backbone was fixed, minimizing all other atoms for additional 5 × 10^3^ steps. No constraints were applied to the final 2.5 × 10^3^ steps. The system was then equilibrated at 310 K in the (N,p,T) ensemble, using rectangular periodic boundary conditions and the particle mesh Ewald approach to evaluate electrostatics (34). All hydrogen bond lengths were constrained using the RATTLE algorithm (35), thus allowing a 2-fs integration timestep. A multiple time-stepping algorithm was utilized, where bonded interactions were evaluated at every timestep, short-range non-bonded interactions were evaluated at every two timesteps, and long-range electrostatic interactions were evaluated at every two timesteps. A cutoff of 14 Å was enforced for the non-bonded calculations, including a switching function at 12 Å. The first 1-ns period was divided into a series of four 2.5 × 10^5^ step runs, where harmonic constraints were employed with 1-fs timesteps to gradually relax the system before the free sampling phase. A harmonic constraint force constant of 4.0 kcal/mol/Å^2^ was applied to protein backbone atoms with scaling factors of 1.0, 0.75, 0.50, and 0.25 for the sequential segments, respectively. Free dynamics were subsequently performed using a 2-fs timestep, for a total of 40 ns for each system. Trajectories were generated on the DataStar machine (San Diego Supercomputing Center, University of California, San Diego, La Jolla, CA, USA) (benchmark time of 0.3 days/ns using 112 parallel processors).

### RMSD-clustering to extract representative MD structures

To generate a reduced, representative set of N1 structures for the CS-Map calculations, RMSD conformational clustering was performed based on a previously reported clustering algorithm (36) as implemented in the rmsdmat2 and cluster2 programs of the gromos++ analysis software (37), part of the gromos05 software for biomolecular simulation (http://www.igc.ethz.ch/GROMOS). The same procedure has been recently applied to the context of protein surface loops (38) and of an improved relaxed complex method for drug design (39).

**Figure 1:**
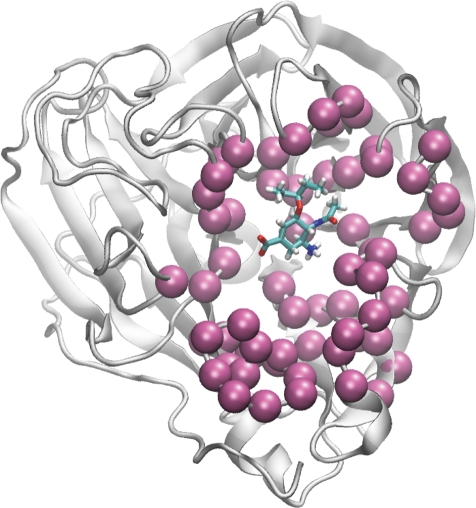
The 62 residues lining the N1 binding site used for RMSD-based clustering are shown with their C_*α*_'s shown in space-filling. They encompass the 150- and 430-loops. Tamiflu is shown bound in the active site, colored by atom-type.

For the apo and oseltamivir-bound simulations, each chain of the tetramer was extracted at 10-ps intervals over the 40-ns simulation. A total of 1.6 × 10^4^ trajectory structures for each simulation were superimposed using all C_α_-atoms to remove overall rotation and translation. The RMSD-clustering was performed on the subset of 62 residues that line the entire binding-site area, which we define here as the binding-site residues: 117–119, 133–138, 146–152, 156, 179, 180, 196–200, 223–228, 243–247, 277, 278, 293, 295, 344–347, 368, 401, 402, and 426–441 (Figure 1). These residues were clustered into batches of similar configurations using the backbone atom-positional RMSD of all atoms (including side chains and hydrogens) as the similarity criterion. A cutoff of 1.3 Å was chosen after evaluation of the dependence of cluster populations against the total number of clusters found for each simulation using a cutoff in the range 1.0–1.5 Å. Next, for both the apo and holo simulations, hydrogen bonding was monitored alternatively using structures from the X-ray crystallographic models, the entire MD ensemble, or the structures belonging to each of the three most populated clusters.

### Computational solvent mapping

**Figure 2:**
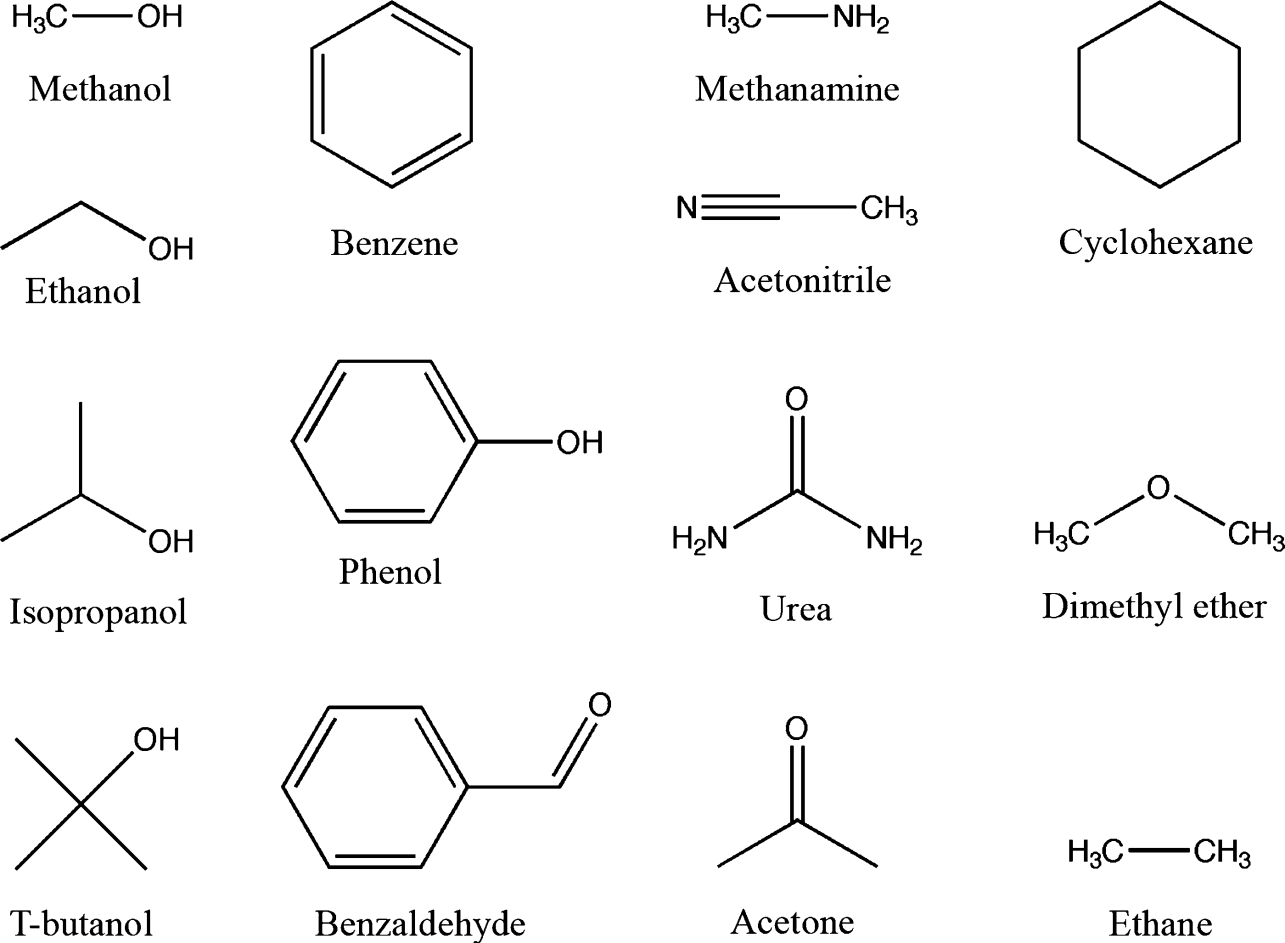
Probe set used for CS-Map.

The CS-Map algorithm is described in the sequence below. Required input is a protein structure, where prior to initiation, any bound ligands and/or water molecules are removed. The 14 organic probes utilized in this study correspond to those employed in the previous hot spot analysis (21) (Figure 2). Mapping simulations were performed on the IBM Blue Gene supercomputer at Boston University, Boston, MA, USA (http://www.ibm.com), where the average CPU time for the first two steps averages 15 min using 220 processors.

#### Rigid body search

For each probe, we generate 222 initial positions on the protein surface along vectors originating from the center of the protein at every 18°. From each of these 222 points, we launch 30 simplex (40) runs with initial simplexes randomly selected around each point, resulting in 6660 docked conformations. Simplex moves are evaluated according to the energy equation:

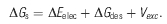

The terms in the equation denote the Coulombic contribution of the electrostatic energy, the desolvation energy, and the excluded volume penalty, respectively. The electrostatic energy (Δ*E*_*e* lec_) is the summation over all probes atoms of the product of the electrostatic field of the solvated protein, denoted as *Φ*, at a particular probe position, determined by a finite-difference Poisson–Boltzmann method (41,42), and the charge *q* of the probe atom. Mathematically, 
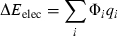
 The desolvation term is calculated in accordance with the atomic contact potential (ACP) model (43), where partial charges are calculated using Quanta (http://www.accelyrs.com). The excluded volume term (*V*_exc_) is set to zero if the probe does not overlap the protein.

#### Refinement of docked conformations

Resulting from the rigid body search are 6660 protein-probe complexes that undergo further minimization. At this stage, a van der Waals energy is included in the free energy calculation Δ*G* = Δ*E*_elec_ + Δ*E*_vdw_ + Δ*G*_des_*. The new desolvation term includes the solute–solvent van der Waals interaction energy. The electrostatic and desolvation terms are calculated using the analytic continuum electrostatic (ACE) model (44); each protein-probe minimization is performed using the Newton–Rhapson method implemented in CHARMM (45). While the protein is held rigid during the minimization, the probe molecules are allowed to be flexible. A maximum of 1000 minimization steps are needed; often fewer steps are required for convergence of Δ*G*.

#### Probe clustering, scoring, and ranking of conformations

Once the probes are minimized in step two, they are clustered based on their residue interactions. Clusters are initiated by seeding with an unclustered probe with the lowest free energy. Next, the remaining unclustered probes are searched to find the probe with the lowest distance score D to the seed probe. The score D is determined by the equation 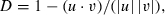 where *u* and *v* are binary strings representing the interactions for each ligand. If this score is below 0.35, then the probe is added to the cluster. The threshold score of 0.35 was chosen such that unclustered probes could be assigned uniquely to one cluster. Once more than two probes are part of the cluster, additional probes are added to the cluster by checking that the average distance score between the new probe and all existing members is below 0.35. If the new probe has an average score above 0.35, then the probe is rejected and a new search begins. Once all probes are checked for admittance to a cluster and no additional probes can be added, a new cluster begins by repeating the process. After initial creation of clusters, probes are re-clustered such that if the average overlap score D of two clusters can be improved by moving a probe from one cluster to the other, then the probe is moved. Subsequent to re-clustering, clusters consisting of less than 20 probes are removed. For each remaining cluster *i*, the probability *p*_*i*_ is calculated based on a partition function *Q*, which is the sum of Boltzmann factors over all conformations *j*: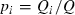 where 
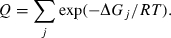
 and *Q*_*i*_ is obtained by summing the Boltzman factors over conformations in the *i*^th^ cluster only. The mean values Δ*G* of free energy of cluster is calculated by the formulation 
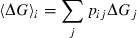
 where 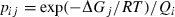


#### Determination of consensus sites

From each of the five lowest free energy clusters for each probe, the lowest free energy conformation is selected and superimposed with all chosen clusters. The locations on the protein where clusters of different probe types aggregate are termed ‘consensus’ sites, and these positions on the protein are considered to be putative binding pockets subject to further evaluation.

## Results and Discussion

### Clustering of MD snapshots to generate a reduced and representative receptor ensemble

To distill the most dominant configurations of N1 from the MD simulations, RMSD-based clustering was performed on snapshots which were extracted from the trajectories for every 10 ps. Although the tetramer N1 was used in the simulations, the clustering analyses were carried out on individual monomer protein chains. Therefore, each 40-ns tetramer simulation yielded the equivalent of four times the monomer sampling (160 ns), and 1.6 × 10^4^ structures for each system were employed in the analyses.

**Figure 3:**
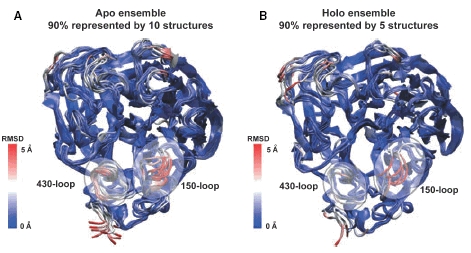
The central member structures of the dominant clusters from the apo and holo molecular dynamics ensembles are shown in A) and B), respectively. Structures are colored by RMSD per residue of each cluster structure to the 2HU4 crystal structure (which is not shown). Note the two most variable regions in the binding site are the 150- and 430-loop regions.

For the apo simulation, the 1.3-Å cutoff resulted in a total of 51 clusters, with 90% of the ensemble represented by 10 clusters (Supplementary Material (SM)Figure 1A). In comparison, the holo simulations revealed an overall less flexible system, as the same cutoff yielded a total of 27 clusters, with 90% of the ensemble being represented by five clusters ((SM) Figure 1B). For both the apo and holo simulations, although the entire binding-site region was used in the clustering, the greatest structural diversity is found in the 150- and 430-loop areas (Figure 3), indicating that these areas are particularly flexible.

To add physical and structural insight to the resulting clusters, a hydrogen-bonding analysis was performed on the 150-loop region (comprised of residues 140–152) for the X-ray crystal structures, over both the entire concatenated trajectory of the four monomers, and the structures within each of the most dominant clusters (Table 1). The crystal structures have five fully formed hydrogen bonds between residues in the 150-loop region: K143–D142, H144–D142, S145–D142, N146–K143, and T147–N146. Hydrogen bond occurrences differ between the apo and holo simulations. Additional hydrogen bonds involving H144–D142 and S145–D142, which are not present in the crystal structures, form transiently throughout the 160 ns of MD.

**Table 1:** Intramolecular hydrogen bonds (averaged over the four monomers in N1) for the 150-loop

H-bond	X-ray structure		Trajectory		First cluster		Second cluster		Third cluster	
					
Donor–Acceptor	2HTY	2HU0	Apo	Holo	Apo	Holo	Apo	Holo	Apo	Holo
K143:N–D142:OD1	100	100	48	53	56	59	43	50	30	31
H144:N–D142:OD2	100	100	74	90	81	97	74	100	52	51
H144:ND1–D142:OD1	–	–	47	–	46	–	44	–	51	–
H144:ND1–D142:OD2	–	–	69	79	84	79	58	100	41	100
S145:N–D142:O	100	100	98	99	94	100	100	96	96	100
S145:OG–D142:O	–	–	69	62	92	97	96	17	32	–
N146:N–K143:O	100	100	70	79	96	97	96	34	–	61
T147:N–N146:OD1	100	100	51	45	–	81	78	–	86	32

The first column shows the hydrogen bonds (donor:atom–acceptor:atom). The second column shows the relative occurrences (in %) for the 150-loop (residues 140–152) based on the crystal structures. Third and fourth columns report corresponding occurrences over the ensemble of structures from the MD simulations. The following columns show occurrences, calculated from structures in only the first, second, and third most-populated conformational clusters. Hydrogen bonds are defined to have a maximum hydrogen-acceptor distance of 3.5 Å and a minimum donor–hydrogen–acceptor angle of 120°. Only hydrogen bonds occurring for at least 10% of the simulation are shown.

In the apo simulations, the most highly populated cluster (representing approximately 20% of the total apo ensemble, SM Figure 1A) is characterized by seven hydrogen bonds in the 150-loop area. Although the hydrogen bond between T147 and N145 is lost, high occupancies of hydrogen bonds in the D142–K143–H144 region contribute to the overall stability of this structure. The 150-loop in the first cluster for the apo simulations is significantly more open than in the ‘open-loop’ crystal structure published in Ref. (8). It is interesting to note that this so-called ‘wide-open’ structure (14) seems to be energetically and structurally stabilized by locally-formed hydrogen bonds. The second most highly populated cluster also accounts for nearly 20% of the ensemble (SM Figure 1A) and the additional stability of the T147–N146 hydrogen bond. Notably, although several hydrogen bonds are less often formed on average, as compared to the first cluster the S145–D142 hydrogen bond is present over the entire cluster set. From a structural standpoint, the second cluster represents the open-loop crystal structure (PDB 2HTY). The third most highly populated cluster is, on an average, lacking the N146–K143 hydrogen bond and exhibits overall a reduced occupancy of hydrogen bonds. This cluster represents nearly 20% of the total apo ensemble, and its central member structure is similar to the closed-loop crystal structure (Figure 3).

In the holo simulations, the most highly populated cluster represents nearly 40% of the overall ensemble and has more hydrogen bonds than the second or third cluster (totally seven hydrogen bonds). These interactions persist throughout the simulations, as indicated by the high percentages for each hydrogen bond (Table 1). Structurally, the most dominant cluster in the holo simulations is similar to the open-loop crystal structure (8). The second cluster, which is structurally similar to the fully-open MD structure, is defined by a loss of the T147–N146 hydrogen bond and a marked reduction in the persistence of the hydrogen bonds between N146–K143 and S145–D142. The second cluster represents slightly more than 20% of the overall holo ensemble. By comparison with the apo simulation, the clustering results suggest that the presence of oseltamivir in the active site tends to decrease the amount of time the 150-loop samples wide open-loop configuration. The third cluster for the holo simulation is structurally similar to the closed-loop conformation and represents approximately 15% of the overall holo ensemble. This cluster of structures is defined by a loss of the S145–D142 hydrogen bond and markedly decreased values for the K143–D142 and H144–D142 hydrogen bonds relative to both the other clusters.

### Mapping analysis of the apo and holo N1 X-ray structures

Using the chemically diverse probe set defined in Figure 2, mapping analyses were performed on the N1 apo and holo structures available from the PDB (2HTY, 2HU0, and 2HU4). It is important to note that although mapping was performed using the entire structures as input, our analyses here are restricted to those consensus sites found within close proximity to the sialic acid binding region. As the apo conformation of N1 is nearly identical to the open holo conformation, here, we only discuss those results obtained for the open and closed holo conformations.

**Figure 4:**
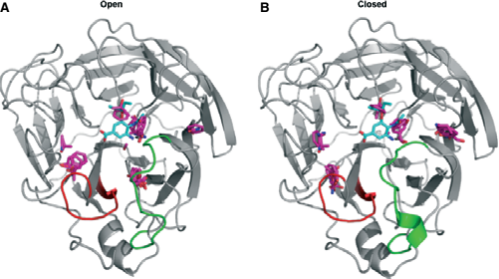
Consensus sites resulting from mapping the A) open structure (PDB 2HU0) and B) closed structure (2HU4) of holo N1. Low energy cluster representatives are colored in magenta, with Tamiflu superimposed and colored by atom-type. In the open and closed conformations a consensus site is observed in the 430-loop region (highlighted in red); however, a consensus site in the 150-loop region (highlighted in green) is formed in open conformation. Consensus sites surrounding the Tamiflu binding region are present in all structures.

Figure 4A represents consensus sites resulting the open N1 structure (2HU0), with cluster representatives colored in magenta, revealing distinct hot spots located in the sialic acid, the 430-loop, and the 150-loop regions of the binding pocket. In Figure 4B, hot spots are distinguishable in the sialic acid and 430-loop binding regions of the closed holo conformation; while in the open holo structure, the disappearance of a consensus site in the 150-loop region is observed upon loop closure. Average rankings of the sialic acid, 430-loop, and 150-loop consensus sites when compared with all others found along the protein surface are fourth, second, and third, respectively. Rankings are determined based on the number of low-energy probe clusters comprising the consensus site.

Based on the direct relationship established between consensus site ranking and druggability in previous studies, we hypothesize that binding regions formed in the 430- and 150-loop regions may provide novel opportunities for lead design. Extension of molecules from the sialic acid binding region into the 430-loop binding region may be more robust to changes in conformation of the 150-loop position than molecules designed to bind within the 150-loop region; however, molecules that stabilize the open confirmation 150-loop may also be a viable approach to lead design.

### Mapping analysis of reduced and representative apo and holo MD ensembles

**Figure 5:**
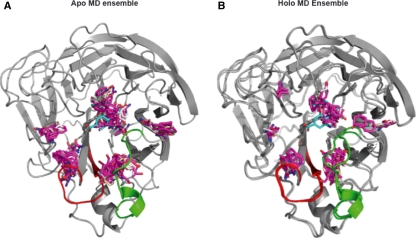
Consensus sites resulting from mapping the A) 10 apo MD structures and B) 5 holo MD structures. Shown all together for each ensemble, low-energy cluster representatives are colored in magenta, and Tamiflu is shown bound in the active site, colored by atom-type. Mapping results for the apo ensemble are superimposed onto the X-ray structure 2HTY, while mapping results for the holo ensemble are superimposed onto the two holo X-ray structures, PDBs 2HU0 and 2HU4. Extended consensus sites in the 430-loop (highlighted in red) and 150-loop (highlighted in green) result from mapping the MD ensembles as compared to the X-ray structures; in the case of holo MD ensemble, a hot spot in the 150-loop is only formed when the MD simulations are used as input. This data demonstrates the utility of using MD-generated conformational ensembles to identify hot spots in flexible loop regions.

Mapping of the 10 apo MD and 5 holo MD representative conformations was performed to explore the effects of receptor flexibility on the formation of hot spots within the 150- and 430-loop binding regions. For each mapping analysis, the entire surface of the protein was utilized by CS-Map. On an average, seven densely-populated (≤10 probe clusters) consensus sites were found for each apo conformation and six were uncovered for each holo conformation. Figure 5 illustrates consensus sites formed within the binding regions of the apo and holo MD ensembles, where in Figure 5A, mapping results for the 10 apo conformations are superimposed onto PDB 2HTY, and in Figure 5B, mapping data for the five holo conformations are superimposed onto PDBs 2HU0 and 2HU4. Similar to mapping results for the X-ray structures, hot spots emerge within the sialic acid, 150- and 430-loop regions of both the apo and holo MD ensembles. For both the apo and holo ensembles, the average rank of consensus sites, in terms of the number of clusters present, located within the sialic acid, 150- and 430-loop regions were fourth, second, and third, respectively. These rankings suggest that the 150- and 430-loop regions represent druggable sites; the relationship between druggability and consensus site ranking is supported by a previous study (21).

**Figure 6:**
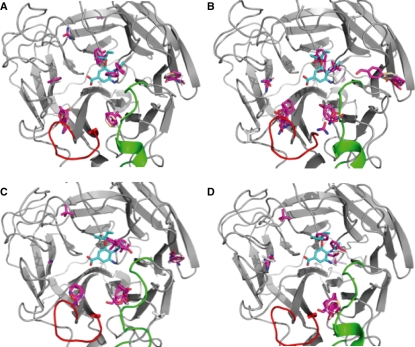
Mapping reveals the effects of loop conformation on hot spot formation within the 430-loop (highlighted in red) and 150-loop (highlighted in green) binding regions. A-B) Loop conformations most resembling the open apo conformation of the 150-loop yield strong hot spots within both the 150-loop and the 430-loop regions. C-D) Further opening of both loops results in an eventual loss of a hot spot within the 430-loop region.

Overall, comparing the mapping results shown in Figure 4, a significant expansion in the volume occupied by probe clusters within the 430- and 150-loop regions is observed for both MD ensembles, suggesting that the loop can be stabilized in multiple conformations that would be amendable to ligand design. In Figure 6, we distinguish the mapping results for the open (Figure 6A and B) and wide-open (Figure 6C and D) holo MD-generated conformations. From Figure 6, we conclude that although a hot spot is found within the 430-loop region of the wide-open conformations, further opening of the loop results in its disappearance.

Table 2 provides statistics on the average sizes, in terms of cluster population, and cluster energy rankings of consensus sites found within each of the three binding regions for the representative MD ensemble structures. Consensus sites were formed in the 150-loop region for 9 of the 10 representative apo conformations; the only apo conformation that did not contain a consensus site in the 150-loop region was the closed conformation of the 150-loop. Similarly, four of the five representative holo conformations contained consensus sites within the 150-loop region with the exception of the closed conformation. Within the 430-loop region, consensus sites were not observed in three of the representative apo conformations and one of the representative holo conformations, which is attributed to structural effects of the wide-open and closed conformations. Based on this data, we hypothesize that hot spots formed within the 150- and 430-loop binding regions are roughly comparable in terms of their ability to bind a variety of probe types with good affinity; however, given the effects of loop dynamics on hot spot formation, it may be more feasible to develop a lead that stably binds the 150-loop region when compared with the 430-loop region.

**Table 2:** Summary of mapping results for the MD ensembles

MD ensemble	Sialic acid	150-loop	430-loop
Apo	7(3)	13(5)	12(3)
Holo	8(2)	11(5)	11(3)

The average number of fragment clusters populating each binding region is reported along with the average energy ranking of each fragment cluster in parentheses.

Although the average affinity of probes for the sialic-acid binding region is also very high (Table 2), the diversity of probes binding in this region is significantly decreased when compared with those binding to both of the loop regions. In particular, only the most polar functional groups in the probe set – namely *t*-butanol, isopropanol, and phenol – bind to the sialic acid binding region, while nearly all probe types, both polar and non-polar, bind with high affinity to the loop regions. This finding suggests that the effects of charge on the bioavailability of N1 inhibitors may be alleviated by the addition of high-affinity, non-polar functional groups that can bind one of the loop regions. We note that such insights would not emerge from the application of CS-Map based on single, static crystal structures only.

As the last part of this analysis, in Table 3, we provide a list of residues predicted to significantly mediate either non-bonded and/or hydrogen-bonded ligand contacts within the sialic acid, 430-loop, and 150-loop binding regions. Using the 10 lowest Boltzmann average free energy probe clusters determined for each holo MD representative, all non-bonded and hydrogen-bonded atomic-level contacts existing between residues of the binding region and the mapped probes were calculated using the program hbplus (http://www.biochem.ucl.ac.uk/bsm/hbplus) (46). Atomic interactions were summed up for each residue and then normalized by the total number of interactions occurring within the binding region. In Table 3, we report only those residues that account for at least 2% of the total atomic-level interactions occurring within the binding region. Residues marked with an asterisk indicate those accounting for greater than 5% of all atomic-level probe-residue interactions. Previous studies have established that protein–probe interactions correlate strongly to known protein–ligand interactions (16,21). In this study, the residues predicted to strongly mediate ligand contacts within the sialic acid binding region – E227, E277, R371, and Y406 – are well-known to be important in the binding of Tamiflu and Relenza, suggesting that those predicted to mediate contacts within the 130- and 430-loop regions – in particular, Q136, D151, I437, K432, and T439 – are likely of importance in the development of a novel class of high-affinity N1 inhibitors.

**Table 3:** Residues predicted to mediate ligand contacts based on CS-Map results

Binding Region	Residues predicted to mediate non-bonded ligand contacts	Residues predicted to mediate hydrogen-bonded ligand contacts
Sialic acid	R118, E119, R224, E227, E277, R371*	E227*, S246, E277*, R371*, Y406*
150-loop	V116, Q136*, V149, D151, R152, S153, P154, R156, W178, S195, G196	Q136*, D151*, R152, R156, W178, S179, G196,
430-loop	I427*, R428, R430, P431, K432*, T439*	S404, R430, K432, T439*

Residues marked with an asterisk (*) indicate the residues that account for greater than 5% of all atomic interactions made by mapped fragments within the binding region. The program hbplus was used to calculate all atomic-level interactions occurring between fragments and residues.

## Conclusions

The influenza virus subtype H5N1 is of great concern because of its high virulence and mutation rate, and some strains have already acquired resistance to the currently available anti-influenza drugs such as Tamiflu and Relenza. Two recent studies, one experimental (8) and the other computational (14), uncovered flexibility within the 150- and 430-loop binding regions of N1, and these findings were conjectured to provide new opportunities for drug design. Here, we examined the effects of this newly discovered receptor flexibility on the emergence of hot spots within these regions through the integration of extensive MD simulations, conformational clustering, and CS-Map. We have identified novel hot spots within flexible binding regions of the N1 neuraminidase and shown that such a clustering procedure significantly improves the efficiency of the CS-Map application.

Few computational methods can account for the effects of receptor flexibility, although its importance is well-established (47–49). Recent examples include the development of inhibitors for MAP kinase p38 (50) and PTP1B (51) that have entered clinical trials and the recently approved HIV integrase inhibitor, raltegravir (marketed as Isentress, Merck, Whitehouse Station, NJ, USA) (52,53). Despite these difficulties, by using a multi-faceted approach, we were able to predict the emergence of stable hot spots within the 150- and 430-loop regions of N1. These results provide further support for the feasibility of developing high-affinity inhibitors capable of binding these newly proposed areas. We hypothesize that a putative novel class of inhibitors, which are able to exploit these new hot spots, may potentially exhibit increased oral bioavailability and be less susceptible to structural mutations in N1 and/or evade existing mutations raised in response to currently available drugs.

## Supplementary material

The following supplementary material is available for this article:

Figure S1 Number of clusters representing the apo (A) and holo (B) simulations at a 1.3 (B) RMSD cutoff vs. percentage of ensemble represented.

This material is available as part of the online article from: http://www.blackwell-synergy.com/doi/abs/10.1111/j.1747-0285.2007.00614.x

(This link will take you to the article abstract).
