# Supplementary material for: Novel Druggable Hot Spots in Avian Influenza Neuraminidase H5N1 Revealed by Computational Solvent Mapping of a Reduced and Representative Receptor Ensemble
Source: Chem Biol Drug Des. 2008 Feb;71(2):106–16. doi: 10.1111/j.1747-0285.2007.00614.x (PMC2438278; doi:10.1111/j.1747-0285.2007.00614.x)
Supplement: Figure S1 — Number of clusters representing the apo (A) and holo (B) simulations at a 1.3 (B) RMSD cutoff vs. percentage of ensemble represented. [file jpp0071-0106-SD1.doc]

**Supporting Information**


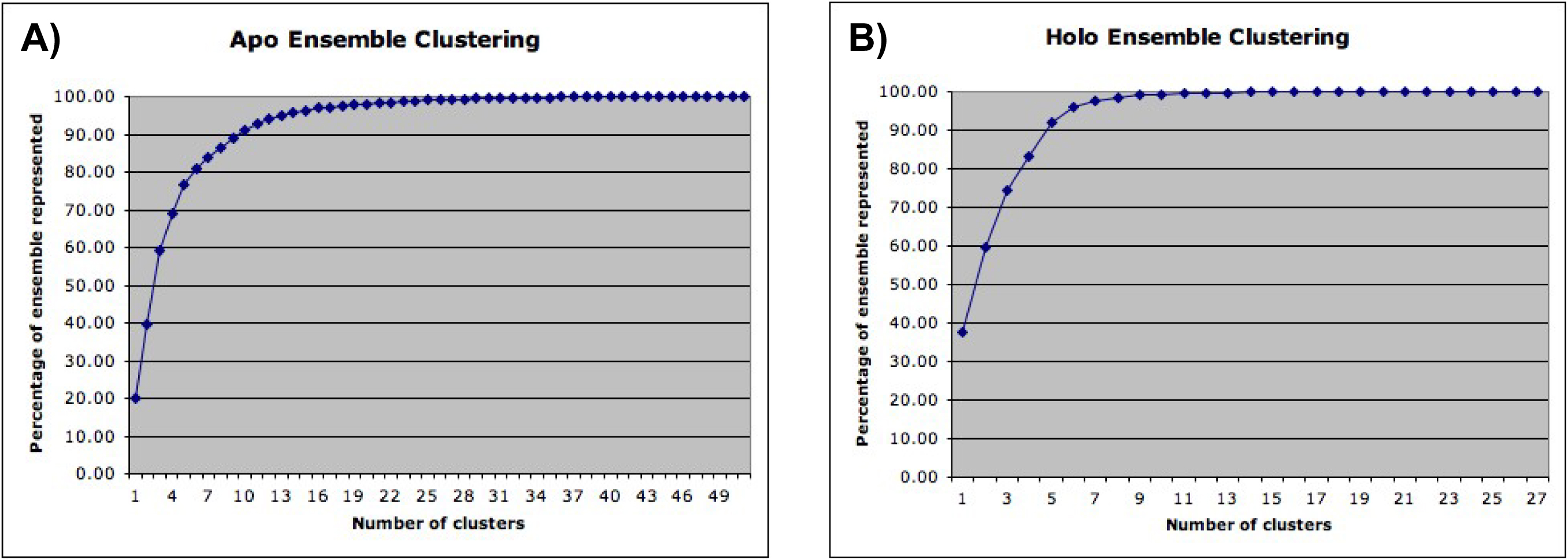


**SI Figure 1:** Number of clusters representing the apo (A) and holo (B) simulations at a 1.3 Å RMSD cutoff *vs.* percentage of ensemble represented.
